# Effect of gardening activities on domains of health: a systematic review and meta-analysis

**DOI:** 10.1186/s12889-025-22263-9

**Published:** 2025-03-22

**Authors:** Feifei Wang, Szilvia Boros

**Affiliations:** 1 School of Public Health, Fudan University, Shanghai, China, Address: Dongan Road 130, Shanghai, 200032 China; 2 Department of Psychology and Health Management, Faculty of Health and Sport Sciences, Széchenyi István University, Address: Egyetem Tér 1, Győr, 9026 Hungary

**Keywords:** Gardening activity, Physical health, General health, Mental health, Horticulture therapy

## Abstract

**Background:**

Gardening activities became increasingly popular in health promotion. The aim of this systematic review was to investigate the effect of gardening-based training or horticultural therapy on domains of health, including mental, physical and general health.

**Method:**

The MEDLINE, PsychINFO, Web of Science, Cochrane, EMBASE, Greenfile, CINAHL, WHO ICTRP, and Clinicaltrials.gov databases were searched from their inception to September 2023. Peer-reviewed, randomized controlled trials (RCTs) or experimental studies with intervention and control groups in English that evaluated the effect of gardening activity or horticultural therapy on health domains. Adult participants living with chronic conditions were selected. Author, year, location, sample size, participant characteristics, study characteristics, type of intervention, measurement time points, measured outcomes, measurements, effect sizes and *p* values were extracted.

**Results:**

Twenty-three studies (*n* = 4535) with 13 RCTs and 10 quasi-experimental studies were included. The participants had a mean age of 54.39 years, and the majority of them were females (63.25%). Types of chronic conditions included physical dysfunction, pain, obesity, anxiety, depression, hypertension, cognition disfunction, etc. The effects of gardening activities were compared with those of the control by categorizing health into three domains: mental health (SMD = -0.31; 95% CI: -0.97, 0.34), physical health (SMD = -0.25; 95% CI: -0.62, 0.11) and general health (SMD = -0.08; 95% CI: -0.20, 0.05).

**Conclusions:**

Gardening-based training programs have a small-to-medium effect on mental health in people living with chronic conditions. Relatively small effects were found for physical health and general health. Future research is recommended to better understand the impact of gardening activities on health.

**Trial registration:**

This systematic review is registered to PROSPERO (https://www.crd.york.ac.uk/prospero/) with registration ID: CRD42024504948.

**Supplementary Information:**

The online version contains supplementary material available at 10.1186/s12889-025-22263-9.

## Background

Chronic disease, also referred as long-term conditions (LTCs), is defined as a persistent or long-lasting human health condition or disease [1]. Common chronic conditions include cardiovascular diseases, arthritis, asthma, cancer, diabetes and viral diseases such as hepatitis C and HIV/AIDS [2]. In recent years, the burden of chronic diseases has increased, as it increases with the crude prevalence of chronic conditions and multimorbidity driven by population aging [3]. In the UK, 24.4% of the population, including 8.3% of school-aged children, 19.6% of working-aged adults, and 66.2% of individuals aged 70 years or more, were at risk of having at least one chronic health condition, 7.1% of which had multimorbidity [4].

The number of management strategies for chronic conditions is increasing given that it is a growing concern worldwide, placing a heavy burden on individuals, families, governments, and health-care systems [5]. Nature-based interventions are increasingly suggested to be beneficial for improving health and well-being for people with a range of health needs. Typically, gardens or gardening comprises a broad spectrum of interventions, activities and outcomes that include plants, the natural environment and living creatures to support people living with chronic health conditions [6].

Increasing attention to therapeutic gardening reflects a broader current interest in the role of nature in enhancing health and well-being. Green spaces are linked to mechanisms and pathways related to public health [7]. Gardening is a leisure activity comprising elements of social engagement, productive activity in a green environment [8]. Over the past few years, several reports have been published from diverse perspectives acknowledging the potential physiological and psychological benefits of exposure to horticulture or gardening [9, 10]. Patients are highly encouraged to engage in gardens, green spaces, parks and the countryside based on a regular basis in the UK [11].

Gardening activities have shown positive effects on comprehensive health outcomes and overrides gardening interventions for health promotion initiatives [12]. Although adequate evidence has suggested that gardening contributes to a healthier lifestyle, creates social opportunities and enables self-development, these results lack the recommended dose and type of gardening activity for therapeutic implementation [13]. Specifically, there is a lack of knowledge regarding how gardening activities influences different health conditions. Furthermore, different types of chronic diseases present specific mechanisms and etiological pathways that could elicit different effects of gardening activities. A precise recommendation requires a better classification of chronic conditions that either attenuate or amplify the disease depending on the cause of health problems.

To date, no previous systematic reviews have specifically explored the effectiveness of gardening in preventing specific domains of health. Thus, we conducted a systematic review and meta-analysis of gardening activities interventions, with our primary aim of exploring their potential effectiveness in domains of health including mental health, physical health and general health. In addition, we aimed to compare the mean differences between the intervention and control groups and reported the significance of gardening interventions for chronic health conditions.

## Methods

We followed the Preferred Reporting Items for Systematic Reviews and Meta-analysis (PRISMA) [14] recommendations for this prospectively registered systematic review (PROSPERO database identifier: CRD42024504948).

### Summary of the search strategy

The search for relevant studies was performed in seven databases (MEDLINE, PsychINFO, Web of Science, Cochrane, EMBASE, Greenfile, Cumulative Index to Nursing and Allied Health Literature (CINAHL)) from the earliest record to 30th September 2023 with an additional search was performed from 30th September 2023 to January 2025. Clinical trial registries, such as the International Clinical Trials Registry Platform (ICTRP) and Clinicaltrials.gov, were also searched for potential trials. The reference lists from previous relevant systematic reviews were checked to identify additional articles that were not included in the database search.

The search used a combination of terms related to gardening activities. All terms were searched as keywords for each database-specific subject heading and MeSH term. The complete search strategy and keywords can be found in the Supplemental Material (eTable 1). Initial records were screened online in the saved lists of each database, and both the title and abstract were fully checked. The search and selection of studies was carried out by two researchers, independently. Initially, the selected records were exported into Excel, and duplicates and screened references were excluded. The final records were subjected to a full-text check, and the detailed reasons for exclusion were documented. Disagreements were resolved by consulting a third researcher.

### Inclusion and exclusion criteria

Studies examined the effect of gardening-related physical activities, including gardening, horticulture, and nature-based outdoor activities were searched. Articles were included if they met the following criteria: (1) randomized controlled trials (RCTs) or single-group before-and-after studies of gardening-related intervention programs. (2) Articles published in peer-reviewed journals and written in English. (3) Participants were adults aged 18 years and older who were living with chronic diseases or conditions (e.g., mental disorder, hypertension, obesity, etc.). (4) Evaluation of gardening activity and/or with comparable groups. If any inclusion criterion was not sufficiently described by the authors, clarification was made by group discussion. Studies published in the form of protocols, dissertations, book chapters, editorials, reviews, case reports, cross-sectional and cohort studies were excluded. Studies involving farmers or individuals involved in agricultural work were not included. The detailed inclusion and exclusion criteria can be found in the Supplemental Material (eTable 2).

### Data extraction

One researcher conducted the data extraction of the included studies using a committee-approved data extraction table. The following information was extracted from the included studies: author, year, location, sample size (total and per group), participant characteristics (e.g., age, % female, etc.), study characteristics (e.g., trial design), type of intervention, measurement time points (i.e., mean and SD at preintervention and postintervention), measured outcomes, measurements used, effect sizes and *p* values.

We define chronic conditions as a broad spectrum of physical and mental dysfunctions [15, 16]. Health is classified into three domains: (1) general health, encompassing quality of life, health status, social connectedness, sleep quality, etc.; (2) mental health, including anxiety, stress, depression, well-being, mood, etc.; and (3) physical health, including blood pressure, cognitive function, HIV care retention, etc. Specific classifications can be found in the Supplemental Material (eTable 1).

### Statistical analysis

Individual effect sizes were extracted for each outcome of interest if the study reported Cohen’s d; otherwise, Cohen’s d was calculated using the pre- and post- intervention mean scores for the treatment and control groups. For studies with multiple outcomes, effect sizes were computed separately. We calculated Cohen’s d and its variance for each outcome using a simplified formula as described by Balbim et al. [17]. A comparison between the simplified and alternative formulas can be found in Supplemental Material eTable 3. We categorized health into three domains, and the overall summary effect sizes were computed separately by meta-analyzing the intervention group and control group. We defined effect sizes as 0.2 for “small”, 0.5 for “medium” and 0.8 for “large”, as suggested by Cohen (1992) [18].

$$\frac{M_{int}-M_{con}}{\sqrt{\left(SD_{int}^2+SD_{con}^2\right)/2}}$$

Heterogeneity was assessed by using the I^2^ statistic. We classified 0%−25% as low heterogeneity, 26%−74% as moderate heterogeneity, and 75%−100% as high heterogeneity [19]. When heterogeneity was low to moderate (< 50%), pooled data were analyzed using a fixed-effects model; conversely, when heterogeneity was moderate to high (> 50%), a random-effects model was employed. The meta-analyses were conducted using RevMan software (version 5.3, Copenhagen).

### Risk of bias and quality assessment

Each included study underwent independent appraisal and was subsequently double-checked for methodological quality using the Cochrane risk of bias tool [20, 21]. The risk of bias for each outcome within a study was categorized as “Low”, “High” or “Unclear” for individual elements from five domains (selection, performance, attrition, reporting, and other) [20]. The quality of evidence for each categorized health outcome was assessed by the Grading of Recommendations, Assessment, Development, and Evaluations (GRADE) framework, which considers four domains (imprecision, inconsistency, indirectness, publication bias). For each pooled health outcome category, the following criteria were used: (1) the number of chronic health conditions in each pooled health outcome (fewer than 5 chronic conditions were downgraded as inconsistent); (2) the number of participants (fewer than 1000 participants were imprecision); (3) risk of bias (downgraded by a threshold of less than 50% heterogeneity as low publication bias); and (4) indirectness (downgraded by participants’ characteristics with regard to age, percentage of females, and reported intervention duration). GRADE has four levels of evidence, also known as certainty in evidence or quality of evidence: very low (i.e., the true effect is probably markedly different from the estimated effect), low (i.e., the true effect might be markedly different from the estimated effect), moderate (i.e., the authors believe that the true effect is probably close to the estimated effect), and high (i.e., the authors have considerable confidence that the true effect is similar to the estimated effect) (see Supplemental Material eTables 4 & 5).

### Equity, diversity and inclusion statement

Our study included all identified trials of gardening activity in people with a broad range of chronic diseases or conditions (Supplemental Material eTable 1). The study population included a spectrum of ages, sexes, demographics and comorbidities. In discussing the generalizability of our results and the limitations of our findings, we acknowledge that this meta-analysis did not purposefully recruit people from marginalized communities.

## Results

### Study identification

**Fig. 1:**
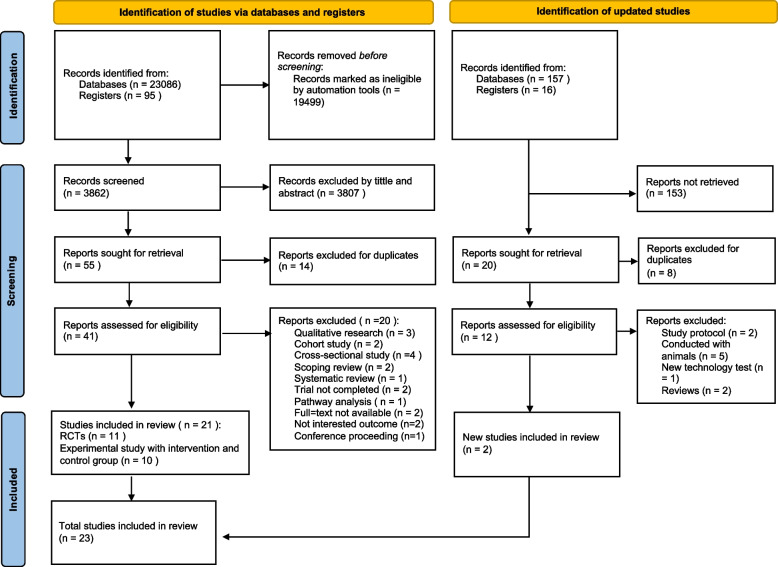
Study selection process for the first round and second round selection. *RCTs: randomized controlled trials*

We adopted the PRISMA diagram to depict the flow of information throughout different phases of this systematic review. Figure 1 illustrates the study selection and screening process flow and maps the number of records identified, included and excluded and the reasons for exclusions. The initial search yielded 23,086 articles, and 95 records were identified, with no additional findings from reference list searches. After employing automation tools and eliminating irrelevant information from each database, 3862 articles were screened by title and abstract, and 3807 articles were excluded by title and abstract. Among the retrieved articles (*n* = 55), 14 duplicated articles were excluded. Twenty articles were excluded due to research design (*n* = 14), research status (*n* = 2), full-text unavailable (*n* = 2), and irrelevant outcome (*n* = 2), finally, 21 out of 41 articles were included in the systematic review and meta-analysis. The second-round search identified 173 records, after excluding studies that did not meet the inclusion criteria, two studies remained. Finally, 23 articles were included in the present systematic review and meta-analysis. The detailed reasons for exclusion are presented in Fig. 1.

### Study characteristics

A summary of the main characteristics of the included studies is provided in the Supplemental Material eTable 6. Among the included studies, thirteen studies were randomized controlled trials, and ten were quasi-experimental studies with intervention and control groups. The sample size ranged from 14 [22] to 381 [23], with a total of 4535 participants included. The participants had a mean age of 54.39 years, and the majority were females (63.25%). The majority of studies were conducted in the U.S. [22–28], four in Taiwan (China) [29–32], two in mainland China [33, 34], and two in Singapore [35, 36]. Single studies were selected from Denmark [37], Norway [38], France [39], the UK [40], the Netherlands [41], South Korea [42], the Dominican Republic [43] and Nicaragua [44].

Health was categorized into three domains: physical health, mental health and general health (summarized in Table 1). The most frequently reported outcomes included depression [30, 35, 36, 42], anxiety [24, 34–36, 42], emotional well-being [22, 26, 28, 31, 36, 40] and cognitive function [33, 35, 36, 39]. Fourteen studies were included in the meta-analysis of gardening activity and mental health [22–24, 29, 32–36, 38, 40, 42]; seven studies were included in the meta-analysis of gardening activity and general health [22, 23, 29, 31, 36, 40, 42]; and five studies were included in the meta-analysis of gardening activity and physical health [33, 35, 36, 39, 44].

**Table 1:** Classification of chronic disease outcomes

Domains	Chronic disease outcomes
General health	Quality of life, General health, Physical functional, Pain, BMI, Satisfaction with life
Mental health	Anxiety, Stress, Depression, Well-being, Mood, Apathy, Mindfulness, Meaning in life, Perceived mattering, Loneliness
Physical health	Systolic blood pressure, Diastolic blood pressure, Cognition function, HIV care retention

*Abbreviation: BMI* body mass index, *HIV* human immunodeficiency virus

### Effects of gardening activity on mental-, physical and general health

**Fig. 2:**
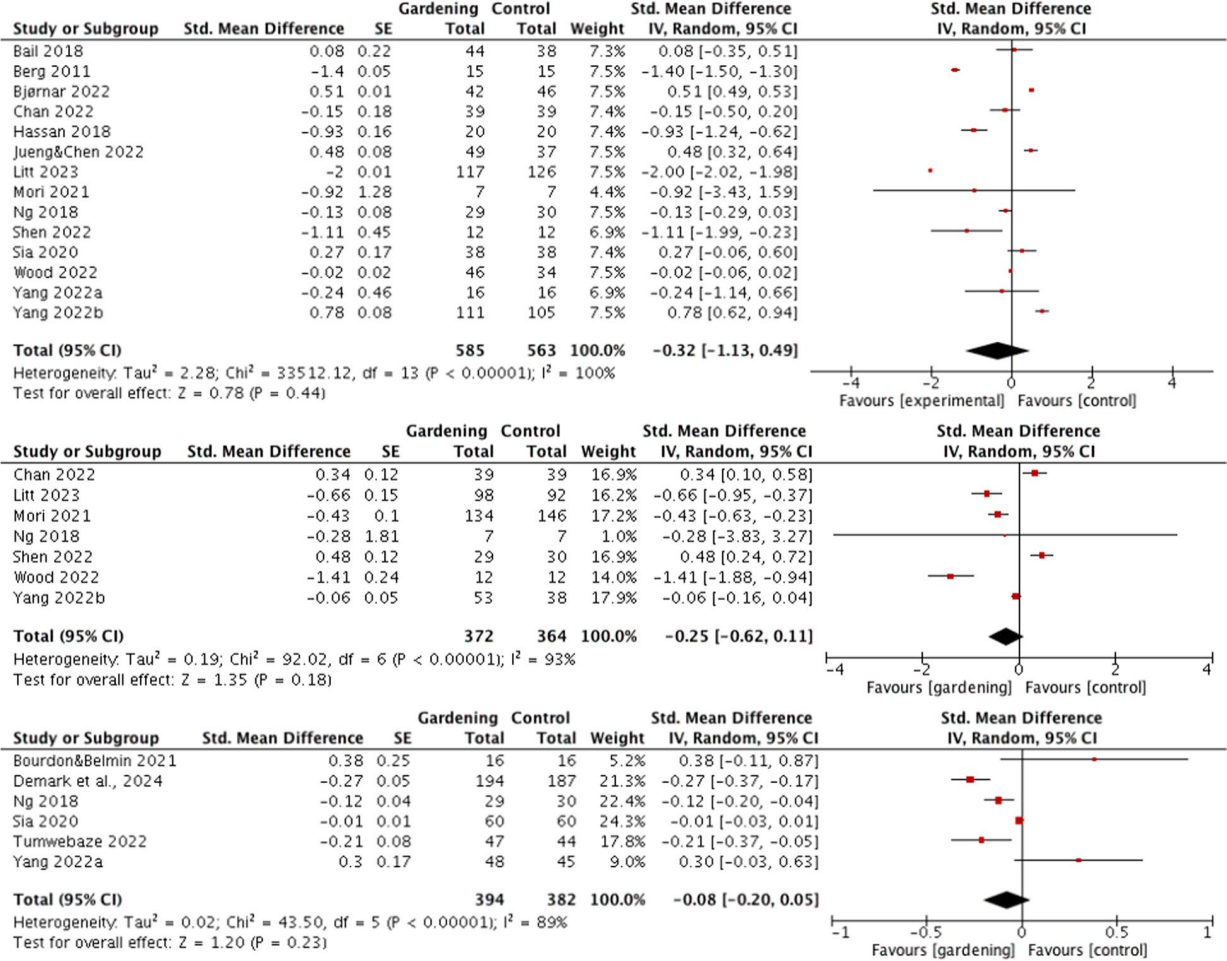
**A**, **B**, **C** Forest-plot of gardening and health domains

The results of the meta-analysis of pooled mental health data from 14 trials investigating gardening interventions and control groups revealed high heterogeneity (100%). With 585 participants in the intervention group and 563 participants in the control group, the overall effect size was relatively low to medium (Z = 0.94, *p* = 0.35), without statistical significance (Fig. 2A). For general health, there were a total of 736 participants in gardening interventions, and for physical health, there were 776 participants. The heterogeneity for both general health (I^2^ = 93%) and physical health (I^2^ = 89%) was relatively high, with overall effect sizes of 1.35 (*p* = 0.18) and 1.20 (*p* = 0.23), respectively (Fig. 2B, C).

Given the high heterogeneity in each subcategory of health outcomes, a random effects model was applied. The meta-analysis showed that gardening activity had a moderate effect on mental health (SMD: −0.31; 95% CI: −0.97, 0.34), a medium-to-low effect on general health (SMD: −0.25; 95% CI: −0.62, 0.11), and a small effect on physical health (SMD: −0.08; 95% CI: −0.20, 0.05). Although the 95% confidence intervals for all these health domains included 0, the overall trend pointed toward a beneficial effect of gardening interventions.

### Sensitivity analysis

We conducted sensitivity analyses of gardening interventions and health outcomes by excluding very low-quality trials. After removing trials rated as “high risk of bias” in at least one domain out of seven domains of the GRADE, the results indicated that gardening intervention is significantly effective for mental health (SMD: −0.82; 95% CI: −1.51, −0.13). Weak evidence was found for general health (SMD: −0.10; 95% CI: −1.16, 0.95) and physical health (SMD:0.06; 95% CI: −0.35, 0.46), with high heterogeneity (I^2^ > 80%). The detailed results can be found in eFigure 1A, B and C.

### Risk of bias and quality assessment

**Fig. 3:**
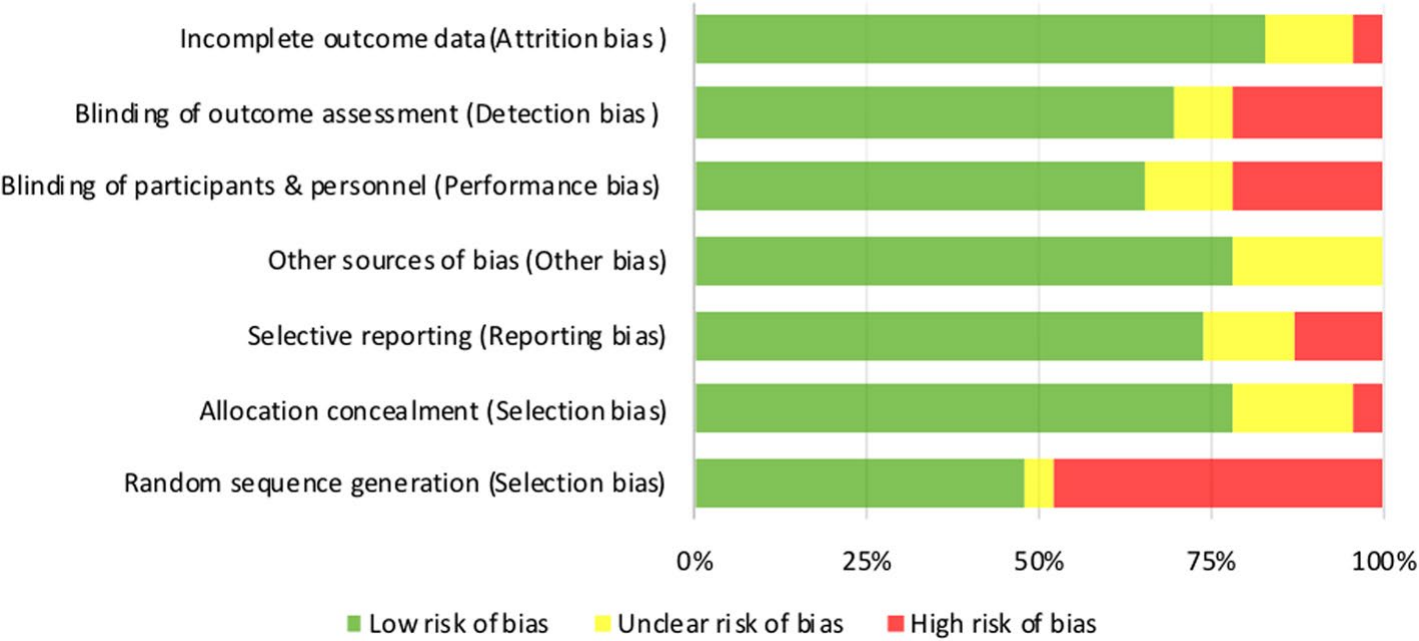
Risk of bias graph. Risk of bias items are presented as percentages across all included studies

There was a minor degree of risk of bias observed in the included studies. The highest potential of bias was related to selection bias, as half of the trials did not adhere to the randomized allocation of participants (50%). Other potential biases included detection bias (22%) and performance bias (35%), which had relatively high percentages of “unclear risk of bias” and “high risk of bias”, respectively, compared with other types of biases (Fig. 3). There was one study obtained more than three “high risk of bias” scores, as their study was conducted in a low-income country where participants engaged in home gardening primarily to improve food security, increasing the risk of selection bias [44]. No other trials received more than three “high risk bias” bias scores for a domain (Supplemental Material eTable 5).

The quality of evidence (GRADE) was rated for three health domains (i.e., mental health, general health, and physical health) (Table 2). The GRADE quality score was rated as moderate, low, or very low for mental health, general health or physical health, respectively, after evaluating inconsistency, indirectness, imprecision and publication bias. Inconsistency and imprecision were found for physical health, and no indirectness was detected for any of the three health outcomes. Detailed information regarding the quality assessment is shown in Table 2.

**Table 2:**
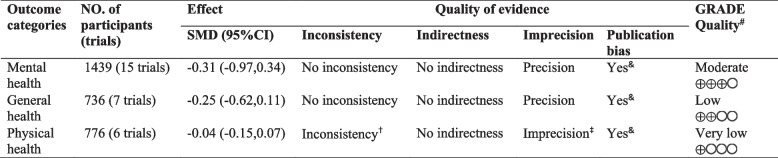
Grading of recommendations, assessment, development, and evaluations (GRADE) summary of findings

^†^Identified chronic health conditions were less than 5 in each category was downgraded for inconsisteny

^‡^With < 1000 participants for each outcome based on characteristics of participants included in each outcome category

^&^Medium to large heterogeneity (I2 > 50%). Calculated by heterogeneity test in meta-analysis

^#^GRADE quality of evidence was categorized into very low, low, moderate considering whether the true effects are markedly different from the estimated effects

## Discussion

### Summary of main findings

We identified twenty-three trials exploring gardening treatments for various chronic conditions that exhibited bias across multiple domains. Gardening interventions were predominantly conducted among elderly adults. Based on the adequate number of reported trials, gardening in general seems to enhance mental health. However, uncertainties exist regarding whether gardening interventions contribute to improved physical health or general health due to the limited number of trials conducted. Additionally, there is uncertainty about which type of gardening activities is most beneficial. A meta-analysis investigating the effects of gardening interventions on mental health, physical health and general health requires more high-quality trials to interpret sound evidence on the impact of gardening interventions on health domains.

### The effect of gardening activities on different health domains in chronic conditions

Gardening activities and horticultural therapies are highlighted for their role in rehabilitation; mediating emotional, cognitive and/or sensory motor functional improvement; increasing social participation; and enhancing health, well-being and life satisfaction [45]. Our meta-analysis on gardening and chronic conditions demonstrated the benefits of gardening, particularly for mental disorders. These results align with previous meta-analyses suggesting a substantial positive effect of gardening on psychological and social health benefits [12]. Our findings, comparing gardening activities and control groups, revealed that gardening interventions are more effective at improving mental health than at improving physical and general health. This outcome is supported by both quantitative and qualitative research demonstrating that gardening has a positive influence on psychological well-being [46, 47].

Gardening activities were most frequently conducted among participants experiencing cognitive decline, such as dementia and Alzheimer's disease, on a global scale [48, 49]. Among the included studies, more than half of the trials were carried out with participants with dementia or community-dwelling elderly adults [25, 29–33, 35, 36, 38, 39, 42]. Gardening activities offer an active lifestyle and preventive healthcare, and serving as non-pharmacological therapy. Additionally, gardening intervention programmes promote social connection for elderly adults, which may enrich their life quality physically and mentally. Gardening intervention is highly encouraged for both community- dwelling older adults and adults in a nursing home (assumed with cognitive impairments). Existing evidence highlights the growing population of individuals living with cognitive decline in the community setting, highlighting the potential for green spaces to facilitate an active community life [50]. Gardens, horticultural programs and neighborhood outdoor environments have been shown to positively impact people living with dementia [51]. Although the amelioration of cognitive decline and well-being may not be fully understood through gardening, the restorative effects of the outdoor activity and environment have been well documented [52].

### Heterogeneity and publication bias

Our results revealed an estimated 31% reduction in mental health issues within the gardening intervention group compared to the control group, with estimated mean differences of 25% and 4% for general health and physical health, respectively. These estimates were slightly lower than those reported in previous meta-analyses, which indicated a 42% difference in psychosocial well-being between the gardening group and the control group [12]. The observed high heterogeneity (I^2^ > 70%, *p* < 0.0001) may be attributed to the diversity of gardening activities, intervention durations, and the broad ethnic background of the participants. This level of heterogeneity aligns with findings from previous systematic reviews meta-analyzing sport- or physical activity-related interventions [53]. Our results seem promising, as the sensitivity analysis showed that after excluding very-low-quality studies, the effectiveness of the gardening intervention became significant for mental health. However, future studies with high-quality designs and comparable participant levels are still needed.

### Strengths and limitations of the included trials

The strengths of this systematic review include the incorporation of a broad range of chronic diseases and/or health conditions, encompassing RCTs and experimental trials with comparable groups. We assessed the risk of bias and conducted a thorough evaluation of the quality of evidence. All reported chronic diseases or conditions from each included study were labeled and categorized into health domains, along with the reported number of participants in the intervention and control groups. Nevertheless, the search strategy did not specify date restrictions, allowing for the maximal inclusion of trials conducted over an extended period. The most recent evidence, which comprehensively searched 14,321 records and included 50 nature-based outdoor activities for mental and physical health, highlighted gardening as the most commonly tested activity (included number of studies: *n* = 16) [54]. In this meta-analysis, we included experimental studies involving both randomized and nonrandomized trials of gardening activities. To the best of our knowledge, we computed the largest pooled number of participants for comparison (number of records: *n* = 23,182; included number of studies: *n* = 21).

The overall quality of the included studies was relatively low to medium, according to the GRADE assessment. However, the risk of bias tool demonstrated a low risk of bias across all domains, with the exception of blinding and randomization of participants. The main source of bias within the included trials was randomization. Given that more than half of the studies were conducted with elderly adults experiencing cognition problems, adhering to randomization principles during intervention trials in real-life communities proved challenging. It is acknowledged that achieving blinding and randomization in physiotherapy and physical intervention trials is difficult [55].

We did not perform subgroup analysis by geographical background, age or sex group, as the majority of participants in the included studies were female older adults, indicating good reliability of the included studies. However, a limitation arises when a high level of attrition is present. High attrition rates can bias the results of meta-analyses [56]. Nonetheless, we minimized the risk of bias by conducting sensitivity analysis excluding trials rated as very low quality using the GRADE tool.

### Limitations of this review

For pragmatic reasons, one researcher assessed the risk of bias for the included studies, and two researchers double-checked the extracted dataset. Additionally, an attempt was made to retrieve all published trials; however, the literature search was limited to publications in the English language, and we did not search for studies published in languages other than English. Due to the absence of trials without biases, the effects of gardening interventions were assessed based on those available, and a meta-analysis was performed on those studies. The search did not exclude studies due to varied gardening intervention strategy and duration. There was a relatively high heterogeneity across the studies. However, it's necessary to note that this heterogeneity was unavoidable considering that gardening interventions can be tailored to different settings and forms, which made it difficult to standardize across all studies. Furthermore, consistency in the direction of the effects of gardening can still provide valuable insights.

## Conclusion

In summary, our meta-analysis suggested that gardening activities interventions have a moderate effect on mental health. The effects of physical health and general health on individuals with chronic conditions are unclear. It is crucial to consider participants’ characteristics when implementing gardening interventions, including clarifying gardening activity types, intensity and duration. Furthermore, more research is needed to elucidate the effects of gardening activities on health.

## Supplementary Information

Supplementary Material 1.

## Data Availability

No datasets were generated or analysed during the current study.

## Abbreviations

SMD: Standard mean difference
MD: Mean difference
CI: Confidence interval
RCTs: Randomized controlled trials
GRADE: Grading of Recommendations, Assessment, Development, and Evaluation
CINAHL: Cumulative Index to Nursing and Allied Health Literature
WHO ICTRP: World Health Organization, International Clinical Trials Registry Platform
CDC: Centers for Disease Control and Prevention
