# Supplementary material for: Effect of gardening activities on domains of health: a systematic review and meta-analysis
Source: BMC Public Health. 2025 Mar 22;25:1102. doi: 10.1186/s12889-025-22263-9 (PMC11929992; doi:10.1186/s12889-025-22263-9)
Supplement: Supplementary file 1 — Supplementary Material 1. [file 12889_2025_22263_MOESM1_ESM.docx]

**Supplemental materials**

**Effect of gardening activities on domains of health: A systematic review and meta-analysis**

| **eTable 1** | Search strategy and keywords |
| --- | --- |
| **eTable 2** | Inclusion and exclusion criteria |
| **eTable 3** | Formula for computing cohen’s d |
| **eTable 4** | Levels of evidence certainty |
| **eTable 5** | Risk of bias assessment for individual study |
| **eTable 6** | Summary table of included studies |
| **eFigure 1A** | Forest-plot of gardening and mental health by excluding very-low quality trials |
| **eFigure 1B** | Forest-plot of gardening and general health by excluding very-low quality trials |
| **eFigure 1C** | Forest-plot of gardening and physical health by excluding very-low quality trials |

**eTable 1** Search strategy and keywords

|  | **Intervention keywords** | **General outcomes** | **Specific outcomes** |
| --- | --- | --- | --- |
| 1 | Gardening | Restoration | Cardiovascular disease |
| 2 | Horticulture | Rehabilitation | Hypertension |
| 3 | Horticultural therapy | Health& Disease | Hypercholesterolemia |
| 4 | Organic Agriculture | Noncommunicable disease | Diabetes |
| 5 | Community Gardening | Chronic disease | Heart disease |
| 6 | Nature-based outdoor activity | Well-being | Cancer |
| 7 |  | Physical health | Obesity |
| 8 |  | Quality of life | Arthritis |
| 9 |  | Life satisfaction | Anxiety |
| 10 |  | Happiness | Depression |
| 11 |  | Mood | Stress |

The combination of the these search terms are: (Gardening OR Horticulture OR “Horticultural therapy” OR “Organic Agriculture” OR “Community Gardening” OR “Nature-based outdoor activity” ) AND (“Restoration” OR “Rehabilitation” OR“Health” OR “Disease” OR “Chronic disease” OR “Well-being” OR “Physical health” OR “Quality of life” OR “Life satisfaction” OR “Happiness” OR Anxiety OR Depression OR Stress OR mood OR “Noncommunicable disease” OR “Cardiovascular disease” OR Hypertension OR Hypercholesterolemia OR Diabetes OR “Heart disease” OR Cancer OR Obesity OR Arthritis)

**eTable 2** Inclusion and exclusion criteria

| **Inclusion criteria** | **Exclusion criteria** |
| --- | --- |
| 1. English language  2. Adult participants  3. RCTs (randomized controlled trials) controlled studies, single group before and after studies of outdoor interventions)  4. Human research  5. Evaluation of gardening activity and/or with comparable groups  6. Timeframe: from inception to 30^th^ September 2023 | 1. Study protocols  2. Non-peer reviewed journal articles such as dissertations/books/editorials  3. Reviews (scoping review, systematic review)  4. Case series or case studies  5. Cross-sectional, cohort study  6. Conducted with farmers or agricultural work |

eTable 3 Formula for computing cohen’s d (1988)

| Simplified formula | Alternative formula | References |
| --- | --- | --- |
| 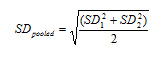  SD1 = standard deviation for group 1;  SD2 = standard deviation for group 2 | 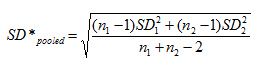  n1 = sample size for group 1;  n2 = sample size for group 2 | Gonick, L. (1993). The Cartoon Guide to Statistics. HarperPerennial.  Klein, G. (2013). The Cartoon Introduction to Statistics. Hill & Wamg.  Kotz, S.; et al., eds. (2006), Encyclopedia of Statistical Sciences, Wiley.  Lindstrom, D. (2010). Schaum’s Easy Outline of Statistics, Second Edition (Schaum’s Easy Outlines) 2nd Edition. McGraw-Hill Education  Website source: https://www.statisticshowto.com/pooled-standard-deviation/ |

eTable 4 Levels of evidence certainty

| **Certainty** | **What it means** |
| --- | --- |
| Very low | The true effect is probably markedly different from the estimated effect |
| Low | The true effect might be markedly different from the estimated effect |
| Moderate | The authors believe that the true effect is probably close to the estimated effect |
| High | The authors have a lot of confidence that the true effect is similar to the estimated effect |

eTable 5 Risk of bias assessment for individual study

| **Studies** | **Random sequence generation (Selection bias)** | **Allocation concealment (Selection bias)** | **Selective reporting (Reporting bias)** | **Other sources of bias**  **(Other bias)** | **Blinding of participants & personnel**  **(Performance bias)** | **Blinding of outcome assessment (Detection bias)** | **Incomplete outcome data**  **(Attrition bias)** |
| --- | --- | --- | --- | --- | --- | --- | --- |
| Bail et al., 2018 | 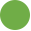 | 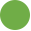 | 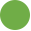 | 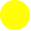 | 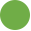 | 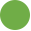 | 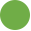 |
| Berg et al., 2011 | 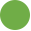 | 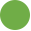 | 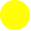 | 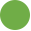 | 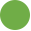 | 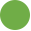 | 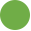 |
| Bjørnar et al., 2022 | 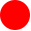 | 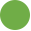 | 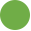 | 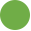 | 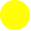 | 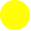 | 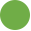 |
| Bourdon & Belmin, 2021 | 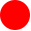 | 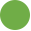 | 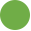 | 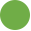 | 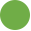 | 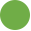 | 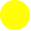 |
| Carney et al., | 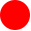 | 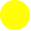 | 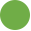 | 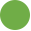 | 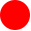 | 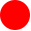 | 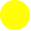 |
| Chan et al., 2022 | 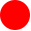 | 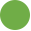 | 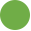 | 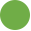 | 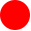 | 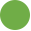 | 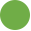 |
| Corazon et al., 2018 | 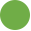 | 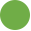 | 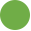 | 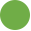 | 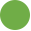 | 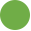 | 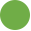 |
| Coringrato et al., 2024 | 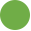 | 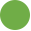 | 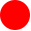 | 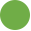 | 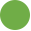 | 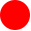 | 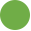 |
| Demark et al., 2024 | 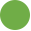 | 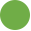 | 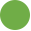 | 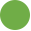 | 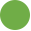 | 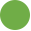 | 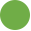 |
| Derose et al., 2023 | 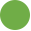 | 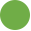 | 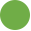 | 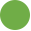 | 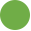 | 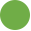 | 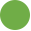 |
| Hassan et al., 2018 | 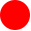 | 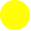 | 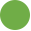 | 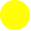 | 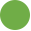 | 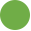 | 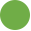 |
| Jueng & Chen, 2022 | 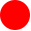 | 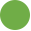 | 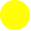 | 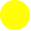 | 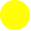 | 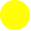 | 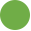 |
| Lin et al., 2020 | 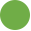 | 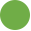 | 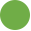 | 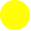 | 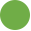 | 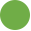 | 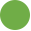 |
| Litt et al., 2023 | 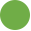 | 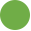 | 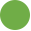 | 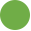 | 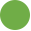 | 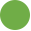 | 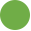 |
| Mori et al., 2021 |  |  |  |  |  |  |  |
| Ng et al., 2018 |  |  |  |  |  |  |  |
| Shen et al., 2022 |  |  |  |  |  |  |  |
| Sia et al., 2020 |  |  |  |  |  |  |  |
| Styck & George,2022 |  |  |  |  |  |  |  |
| Tumwebaze et al., 2022 |  |  |  |  |  |  |  |
| Wood et al., 2022 |  |  |  |  |  |  |  |
| Yang et al., 2022a |  |  |  |  |  |  |  |
| Yang et al., 2022b |  |  |  |  |  |  |  |

Green: Low risk of bias; Yellow: Unclear risk of bias; Red: High risk of bias

eTable 6 Summarized characteristics of included studies

| **Author/year** | **Location** | **Trial design** | **Type of participants** | **Gardening intervention** | **Health outcome** | **Measurements used** |
| --- | --- | --- | --- | --- | --- | --- |
| Bail et al., 2018 | USA | A randomized controlled trial | Breast cancer survivors | Planning, planting, and maintaining gardens | Emotional wellbeing | 36-item Short-Form Health Survey (SF-36) |
| Berg et al., 2011 | The Netherlands | A Randomized Controlled Trial | Members of the allotment complex ‘Amstelglorie’ in Amsterdam | Outdoor gardening activities | Stress | Dutch translation of the Positive and Negative Affect Schedule (PANAS) |
| Bjørnar et al., 2022 | Norway | A quasi-experimental design with comparable groups | People with dementia | Farm-based day care services (FDCs) | Emotional well-being, mood | Maastricht Electronic Daily Life Observation-tool (MEDLO), self-administrated survey |
| Bourdon & Belmin, 2021 | France | Multi-center cluster-controlled pilot randomized contrial tiral | Residents with dementia at nursing homes | Conventional sensory garden visiting and enriched garden visiting | Cognitive function, independence for activities of daily living, risk of falls | Cognitive function (MMSE), independence for activities of daily living (ADL) and risk of falls (unipodal stance and timed up and go – (TUG)) |
| Carney et al., | USA | A quasi-experimental design (pre- post- intervention) | Community-based participants from 21 to 78 years | Program is diverse and historically rooted in agriculture, particularly apple, pear and cherry orchards | Mental health& well-being | Pre-survey and Post-survey questions |
| Chan et al., 2022 | Taiwan | A quasi-experimental design with intervention and control groups | Community-dwelling older adults | Horticultural activities | Quality of life, perceived stress | Structured questionnaires, including WHOQOL-BREF TW, Perceived Stress Scale, and the visuospatial working memory test, were used to collect data. |
| Corazon et al., 2018 | Denmark | A randomized controlled trial | People in sick leave | Nacadia® Nature-Based Therapy | General health | Cognitive Behavioral Therapy StreSS, documents services provided by a GP (face-to-face consultation with the GP, and telephone or online consultation). |
| Demark et al., 2024 | USA | A Randomized Controlled Trial | Mean age of participants was 69.8 years. | One-year, home-based vegetable gardening intervention providing gardening supplies and mentorship by cooperative extension–certified master gardeners to plant and maintain spring, summer, and fall gardens. Waitlisted participants received the identical intervention after 12 months. | Physical function | 36-Item Short Form Health Survey [SF-36] |
| Coringrato et al., 2024 | USA | A Randomized Controlled Trial | Participants were: 1) not having gardened in the last two years, 2) being 18 years of age or older, and 3) the ability to give consent in English or Spanish | The trial collected data from 2017 through 2020 and examined whether community garden participation improved diet, physical activity, and body mass index, and reduced perceived stress and anxiety. Study participants were treated similarly to community gardeners. | Diet, physical activity, and body mass index, and reduced perceived stress and anxiety | Process evaluation data were collected via semi-structured interviews with participants, direct observation of community garden sites, and surveying of participants to assess reach, fidelity, and acceptability. |
| Derose et al., 2023 | Dominican Republic | A Pilot Cluster Randomized Controlled Trial | Adults with HIV | Urban gardening and Garden-Based Nutrition and Cooking Workshop | Detectable viral load, HIV care retention | Hospital records |
| Hassan et al., 2018 | Mainland China | A Randomized Controlled Trial | Chinese older women experiencing psychological stresses and depression | A transplanting activity using soil with plants (yellow chrysanthemum) | Anxiety, blood pressure | State-Trait Anxiety Inventory (STAI),Sphygmomanometer Omron (HEM-7011; Omron, China) |
| Jueng & Chen, 2022 | Taiwan | A quasi-experimental research design with intervention and control groups | Elderly living in long-term care facilities， aged 65 years or above | Horticultural activities (indoor desktop gardening) | Cognitive function, depression | Mini-mental status examination (MMSE) geriatric depression scale (GDS)-15 |
| Lin et al., 2020 | Taiwan | A quasi-experimental design with intervention and control groups | Long-term care facility residents who were over 65 years old | A combination of 3D virtual reality and hands-on horticultural therapy | Health status, meaning in life, perceived mattering, loneliness, and depression | 12-item Chinese Health Questionnaire (CHQ-12), Purpose in Life survey, Perceived mattering was adapted from the General Mattering Scale, The short-form UCLA (University of California, Los Angeles) Loneliness Scale (ULS-6), short-form of the Geriatric Depression Scale (GDS-15) |
| Litt et al., 2023 | USA | Observer-blind, randomized, controlled trial | Adults aged 18 years or older, and had not gardened in the past 2 years. | Introductory gardening courses, and planting | Moderate-to-vigorous physical activity, sedentary time, BMI, and waist circumference， stress, anxiety | Health surveys, including perceived measures of stress and anxiety, accelerometry, dietary interviews, and percived stress scale (PSS-10), Generalised Anxiety Disorder Assessment (GAD-7) |
| Mori et al., 2021 | USA | A quasi-experimental design (pre- post- intervention) | Females with cancer who presented with chronic pain for longer than 3 months | Therapeutic horticulture program | Quality of life (QOL) and physical, social, and emotional functioning | Brief Pain Inventory short form (BPI-SF), the 36-Item Short Form Survey (SF-36), and the Functional Fitness Test (FFT), Interview and Focus Group Discussions |
| Ng et al., 2018 | Singapore | A Randomized Controlled Trial | Older adults have a minimum Montreal Cognitive Assessment (MoCA) score of 22 | Horticultural therapy ranging from indoor gardening, growing, maintaining and harvesting vegetables and herbs | Cognitive functions, depression, anxiety, psychological well-being, social connectedness and satisfaction with life | Montreal Cognitive Assessment (MoCA), Zung Self-Rating Depression Scale (SDS), and the Zung Self-Rating Anxiety Scale (SAS) and the Ryff’s Scales of Psychological Well-Being, Friendship Scale and Satisfaction with Life Scale. |
| Shen et al., 2022 | Taiwan | A quasi-experimental design (pre- post- intervention) | Participants aged 70-93 with day-care services | Asia Pacific Association of Therapeutic Horticulture | Satisfaction with life, mental health | Satisfaction with Life Scale (SWLS), Warwick–Edinburgh Mental Well-Being Scale (WEMWBS) |
| Sia et al., 2020 | Singapore | A quasi-experimental design (pre- post- intervention) | Elderly participants from three senior day care centers | Horticultural-based activity and nature-art activity. | Cognitive function, depression, anxiety | Mini Mental State Exam (MMSE), Zung depression scale, Zung anxiety scale |
| Styck & George,2022 | USA | A Cluster-Randomized Pilot Study | People with dementia aged greater than 50 years old | Donate vegetables and harvested produce | Quality of life with specific focus on sense of purpose in life | Semi-structured, qualitative interviews |
| Tumwebaze et al., 2022 | Nicaragua | A pilot randomized controlled trial | Participants who received training from JustHope, Inc. | Home gardening | Systolic blood pressure, diastolic blood pressure | National Health and Nutrition Examination Survey, Omron BP710N3 Series Upper Arm Blood Pressure Monitor |
| Wood et al., 2022 | UK | A quasi-experimental design with intervention and control groups | Adults (aged 18 years+) | Therapeutic community gardening with various gardening activities including sowing seeds, potting, and general garden maintenance | Wellbeing and mental health | short form Warwick Edinburgh Mental Wellbeing Scale (SWEMWBS), six-item version of the De Jong Gierveld loneliness scale, satisfaction with life scale |
| Yang et al., 2022a | Mainland China | A pilot randomized controlled trial | Nursing home residents with dementia | Horticultural therapy (planting, handicraft, diet) | Apathy, cognitive function, functional capacity | Apathy Evaluation Scaleinformant version (AES-I), Quality of Life in Alzheimer’s disease (QoL-AD) scale, and Barthel Index (BI) |
| Yang et al., 2022b | South Korea | Longitudinal and prospective design, quasi-experimental study | Participants were recruited from local community centers, hospitals, senior welfare centers or dementia daycare centers, community mental health centers, and special schools | Therapeutic gardening program such as observing plants, creating a garden, and taking a walk in the garden. | Mental health and well-being | Mental Health Screening Tool for Depressive Disorders, Mental Health Screening Tool for Anxiety Disorders, Engagement in Daily Activity Scale, brief version of World Health Organization Quality of Life, and Mindful Attention Awareness Scale |

eFigure 1 Forest-plot of gardening activity and health by excluding very-low quality trials
